# Estrogen attenuates *AGTR1* expression to reduce pancreatic β-cell death from high glucose

**DOI:** 10.1038/s41598-017-15237-4

**Published:** 2017-11-30

**Authors:** Suwattanee Kooptiwut, Keerati Wanchai, Namoiy Semprasert, Chatchawan Srisawat, Pa-thai Yenchitsomanus

**Affiliations:** 1 Department of Physiology, Faculty of Medicine, Siriraj Hospital, Mahidol University, Bangkok, 10700 Thailand; 2 Department of Biochemistry, Faculty of Medicine, Siriraj Hospital Mahidol University, Bangkok, 10700 Thailand; 3 Department of Research and Development (Division of Molecular Medicine), Faculty of Medicine, Siriraj Hospital, Mahidol University, Bangkok, 10700 Thailand

## Abstract

Chronic exposure of pancreatic β-cells to high glucose levels results in β-cell dysfunction and death. These effects can be protected by estrogen. The local pancreatic renin-angiotensin system (RAS) has been shown as a novel pathological pathway of high-glucose-induced cell death. The effect of estrogen on pancreatic RAS is still unknown. This study examines whether estrogen protects against pancreatic β-cell death caused by glucotoxicity via a decrease in the pancreatic β-cell RAS pathway. When INS-1 cells were cultured in a high glucose medium, cell death was significantly higher than when the cells were cultured in a basal glucose medium; similarly, there were also higher levels of *AGTR1 and p47* ^*ph*^ *°* ^*x*^ mRNA, and protein expression. Moreover, the addition of 10^−8^ M 17β-estradiol to INS-1 cells cultured in a high glucose medium markedly reduced cell death, *AGTR1* and *p47* ^*ph*^ *°* ^*x*^ mRNA levels, and protein expression. Similar results were demonstrated in the pancreatic islets. The presence of 10^−8^ M 17β-estradiol, losartan, or a combination of both, in a high glucose medium had similar levels of reduction of *p47* ^*ph*^ *°* ^*x*^ mRNA and protein expression, compared with those cultured in high glucose. Taken together, estrogen protected pancreatic β-cells from high-glucose-induced cell death by reducing the *AGTR1* pathway.

## Introduction

Chronic exposure of pancreatic β-cells to high glucose levels causes cellular dysfunction; the resulting β-cell impairment reduces insulin production, thereby causing hyperglycemia^1^. Chronic hyperglycemia and impaired pancreatic β-cell function eventually lead to β-cell death^2^. This condition is known as glucotoxicity. The mechanisms that cause pancreatic β-cell glucose toxicity have not been fully elucidated; however, it has been hypothesized that oxidative stress is a central mechanism for glucose toxicity and pancreatic β-cell damage^3^. Oxidative stress is a condition that results from reactive oxygen species (ROS) generation^4^. ROS is produced by several pathways, including the mitochondrial electron transport system^5^, advanced glycation end-product formation, and glucose autoxidation^6^. The pancreatic β-cell renin-angiotensin system (RAS) is another pathway that induces ROS production through Nicotinamide adenine dinucleotide phosphate-oxidase (NADPH oxidase) complexes^7^. Culturing pancreatic β-cells under high glucose increased angiotensin II receptor (AGTR) mRNA levels and protein expression, which induced the formation of NADPH oxidase complexes^7^. Altogether, the evidence suggests that the pancreatic β-cell RAS plays a role in pancreatic β-cell apoptosis.

The local RAS has been shown to be involved in the pathophysiology of several organs, including the liver and pancreas, while the systemic RAS has been shown to control blood pressure and fluid homeostasis^8^. The pancreatic β-cell RAS component enzymes – including renin, angiotensinogen, and angiotensin converting enzyme (ACE) – are found in the pancreatic acinar and islet cells of both human and murine pancreatic islets^9^. *AGTR1* and *AGTR2* are expressed in different pancreatic islet cell types: *AGTR1* is expressed by the pancreatic β-cells, while *AGTR2* is expressed by the pancreatic α- and δ-cells. While the local acinar cell RAS regulates the exocrine function, the local islet cell RAS regulates glucose-induced insulin secretion^7^. RAS inhibitors, ACE inhibitors or angiotensin receptor blockers (ARB) prevent type 2 diabetes both in humans and animals^10,11^. ARB-mediated type 2 diabetes protection is supported by cell line experiments in which the *AGTR1* blocker increased insulin secretion and proinsulin synthesis^12^.

Estrogen is a steroid hormone that plays an important role in the female reproductive system. Estrogen also regulates glucose homeostasis by improving insulin sensitivity, increasing glucose-stimulated insulin secretion, and increasing glucose transporter expression^13^. Additionally, estrogen replacement in post-menopausal women decreased type 2 diabetes risk^14^. In our previous study, we showed that estrogen treatment improved glucose-stimulated insulin secretion from mouse pancreatic islets that were cultured in a high glucose medium^15^. The role of estrogen on *AGTR1* expression has been examined in several tissues. In ovariectomized rats, *AGTR1* expression was increased in aortic tissue and cultured vascular smooth muscle cells^16^. Estrogen decreased the *AGTR1* expression by inhibiting *AGTR1* translation in the rat adrenal cortex while decreasing *AGTR1* transcription in the pituitary gland^17^. Conversely, estrogen increased cardiac *AGTR1* expression in ovariectomized rats^18^. Thus, the effects of estrogen on *AGTR1* expression are cell type- or tissue-specific. However, the effects of estrogen on the pancreatic β-cell *AGTR1* pathway are not known.

We hypothesized that high glucose enhances pancreatic *AGTR1* expression, which in turn induces pancreatic β-cell apoptosis. Estrogen or an *AGTR1* inhibitor might protect pancreatic β-cells from glucotoxicity by decreasing the pancreatic *AGTR1* pathway. Therefore, the current study investigated the role of estrogen or an *AGTR1* inhibitor on pancreatic β-cell apoptosis, *AGTR1* and NADPH oxidase expression in pancreatic β-cells cultured under high glucose conditions.

## Materials and Methods

### INS-1 cell culture

INS-1 cells were cultured in RPMI 1640 containing 11.1 mM glucose, supplemented with 10% fetal calf serum, 100 U/ml penicillin and 100 μg/ml streptomycin, at 37 °C in humidified air containing 5% CO_2_. The medium was changed every 2 days.

### Animals

The work using animals was approved by Siriraj Animal Care and Use Committee (SI-AUCC). Male ICR outbred 8–12 weeks mice were purchased from the National Laboratory Animal Center, Mahidol University, Bangkok, Thailand. Mice were kept at in a 12-h light/dark cycle environment at 25 ± 2 °C.

### Mouse pancreatic islet isolation

Pancreatic islets were isolated by collagenase digestion by using a modified method of Lacy and Kostianovsky^19^, and Gotoh^20^. Briefly, pancreases were infused with collagenase-P and digested at 37 °C. Islets were separated by using histopaque gradient, and manually picked under a stereomicroscope. All methods were carried out in accordance with ACUC guidelines. The animal experimentation protocol was approved by the Institutional Animal Care and Use Committee, Faculty of Medicine Siriraj Hospital, Mahidol University (Approval No: SI-ACUP 002/2553).

Isolated islets were cultured for 24 hours and the medium was changed to basal or high glucose with or without 10^−8^ M 17β-estradiol for 10 days. Then, mRNA and protein were extracted to perform real-time RT-PCR and Western blot analysis.

### Propidium iodide (PI) staining

INS-1 cells were plated into 6-well plates for 24 h. The medium was replaced with one containing either the basal glucose level (11.1 mM) or a high glucose level (40 mM), with or without 10^−8^ M 17β-estradiol, and cells were further incubated for 72 h. After incubation, the cells were trypsinized and washed. Ice-cold, 70% ethanol was then added to the cells and gently mixed by vortexing. The cells were kept at −20 °C for 1 h. After incubation, the cells were pelleted by centrifugation, and resuspended in 100 μl PBS with RNase. The cells were then transferred to flow cytometry tubes, and PI was added immediately before injecting the cells into a FACScan (Beckton Dickinson, USA) for flow cytometric analysis. The sub-G_1_ DNA content histogram was estimated.

### Caspase 3 activity assay

INS-1 cells were cultured either in normal or high glucose RPMI 1640 media, with or without 10^−8^ M 17 β-estradiol, for 72 h; caspase 3 activity was determined using a caspase 3 colorimetric protease assay (Invitrogen, USA). The assay was performed following the manufacturer’s protocol. Briefly, cells were lysed to obtain protein. The protein concentrations were determined. Fifty μg of protein samples were added with 2X reaction buffer and DEVD-*p*NA substrate, and then incubated at 37 °C for 2 hours in the dark. After incubation, the caspase 3 activities were determined by spectrophotometer at a wavelength of 400 nm.

### MTT assay

INS-1 cells were cultured either in normal or high glucose RPMI 1640 media, with or without 10^−8^ M 17 β-estradiol and/or 1 µM losartan, in a 96-well plate for 72 h. Cell viability was determined using a colorimetric MTT assay. In brief, 5 mg/ml 3-(4,5-dimethylthiazol-2-yl)-2,5-diphenyl tetrazolium bromide thiazolyl blue (MTT) was added to the cells, and incubated at 37 °C, 5% CO_2_, for 4 h. The medium was removed, and 0.1 N HCl acidic isopropanol was added to each well and mixed. The cells were then incubated at 37 °C, 5% CO_2_, for 1 h. Absorbance was measured at 570 and 650 nm, using a PowerWave^TM^ microplate scanning spectrophotometer (BIO-TEK, USA.) Cell viability was calculated from the average corrected 570 nm absorbance value using the following equation:

$$ \% \,{\rm{cell}}\,{\rm{viability}}=\frac{({\rm{corrected}}\,{\rm{OD}}\,570\,-\,{\rm{corrected}}\,{\rm{OD}}\,650){\rm{of}}\,{\rm{sample}}}{({\rm{corrected}}\,{\rm{OD}}\,570\,-\,{\rm{corrected}}\,{\rm{OD}}\,650){\rm{of}}\,{\rm{control}}}\times 100$$

### Measurement of intracellular superoxide generation

The superoxide production was detected by Nitroblue tetrazolium (NBT) assay. Briefly, INS-1 cells were cultured in 11.1 mM or 40 mM glucose, with or without 10^−8^ M 17 β-estradiol, for 72 h. After incubation, the cells were incubated with NBT for 90 minutes. The cells were then lysed in potassium hydroxide (KOH) and dissolved in DMSO. The amount of superoxide production was measured by optical density (OD) at a wavelength of 630 nm, using a PowerWave microplate scanning spectrophotometer (BIO-TEK, USA).

### RNA isolation and real time-polymerase chain reaction (RT-PCR)

The total RNA was extracted from INS-1 cells using a High Pure RNA Isolation Kit (Roche Diagnostic Corporation, USA) according to the manufacturer’s instructions. The total RNA concentration was measured with a ND-1000 Spectrophotometer (Nanodrop, USA). The first strand complementary DNA (cDNA) was generated from 1 μg total RNA, using SuperScript III reverse transcriptase (RT) and random hexamer primers (Invitrogen, USA), and in accordance with the manufacturer’s instructions. The primers were synthesized by Sigma-Aldrich (Sigma-Aldrich, USA). Real-time PCR primers for *AGTR1* and *p47* ^*phox*^ were used, as described in a previous study^12^. The β-Actin primers were 5′-ATG AAG TGT GACGTTGACATCGTC-3′ and 5′-CCTAGAAGCATTTGCGGTGCACGATG-3′. A real-time PCR was performed to amplify specific cDNA sequences with the Brilliant^®^ II SYBR^®^ Green QPCR Master Mix (Agilent Technologies, USA). The PCR programs were 95 °C incubation for 10 min, 95 °C denaturation for 30 sec, 57 °C annealing for 15 sec, and 72 °C extension for 30 sec. The gene expression was calculated by the 2^−∆∆Ct^ method, and was presented as fold change compared with the control.

### Whole cell protein extraction

The total protein was extracted from INS-1 cells or mouse pancreatic islets that had been treated under experimental conditions for 48 h with a radioimmunoprecipitation assay (RIPA) buffer containing Halt Protease Inhibitor Cocktail (Pierce, USA). Briefly, cold RIPA buffer was added to the culture plate and shaken on ice for 5 minutes. Cell lysates were then transferred to Eppendorf tubes, and centrifuged to obtain whole cell proteins. The protein concentration was measured with a MicroBCA^TM^ protein reagent kit.

### Western blot analysis

The total protein (50 µg) was loaded onto a polyacrylamide gel and separated by electrophoresis. The separated proteins were then transferred onto a nylon membrane with an electroblotting apparatus. After that, the membrane was blocked with 5% skimmed milk. The membrane was incubated with one of the following primary antibodies: rabbit polyclonal anti-*AGTR1* (Santa Cruz Biotechnology, USA), goat polyclonal anti-*p47* ^*phox*^ (Santa Cruz Biotechnology, USA), or mouse monoclonal anti-β-actin (Santa Cruz Biotechnology, USA), overnight at 4 °C. Then, the membrane was washed and incubated with HRP-conjugated secondary antibody (Santa Cruz Biotechnology, USA) at room temperature in the dark for 1 h. The protein bands were detected with SuperSignal Pico Chemiluminescence Luminol Substrate (Pierce, USA) and X-ray film exposure, followed by scanning and quantification with ImageJ software.

### Statistical analysis

The data were analyzed with SPSS software, version 17 (SPSS Inc., USA), and expressed as the mean ± standard deviation (S.D). Differences between the groups were determined by one-way ANOVA followed by Tukey’s post-hoc test. A *p*-value of less than 0.05 was considered to be statistically significant.

## Results

### Estrogen reduced cell death of INS-1 cells cultured in high glucose medium

**Figure 1:**
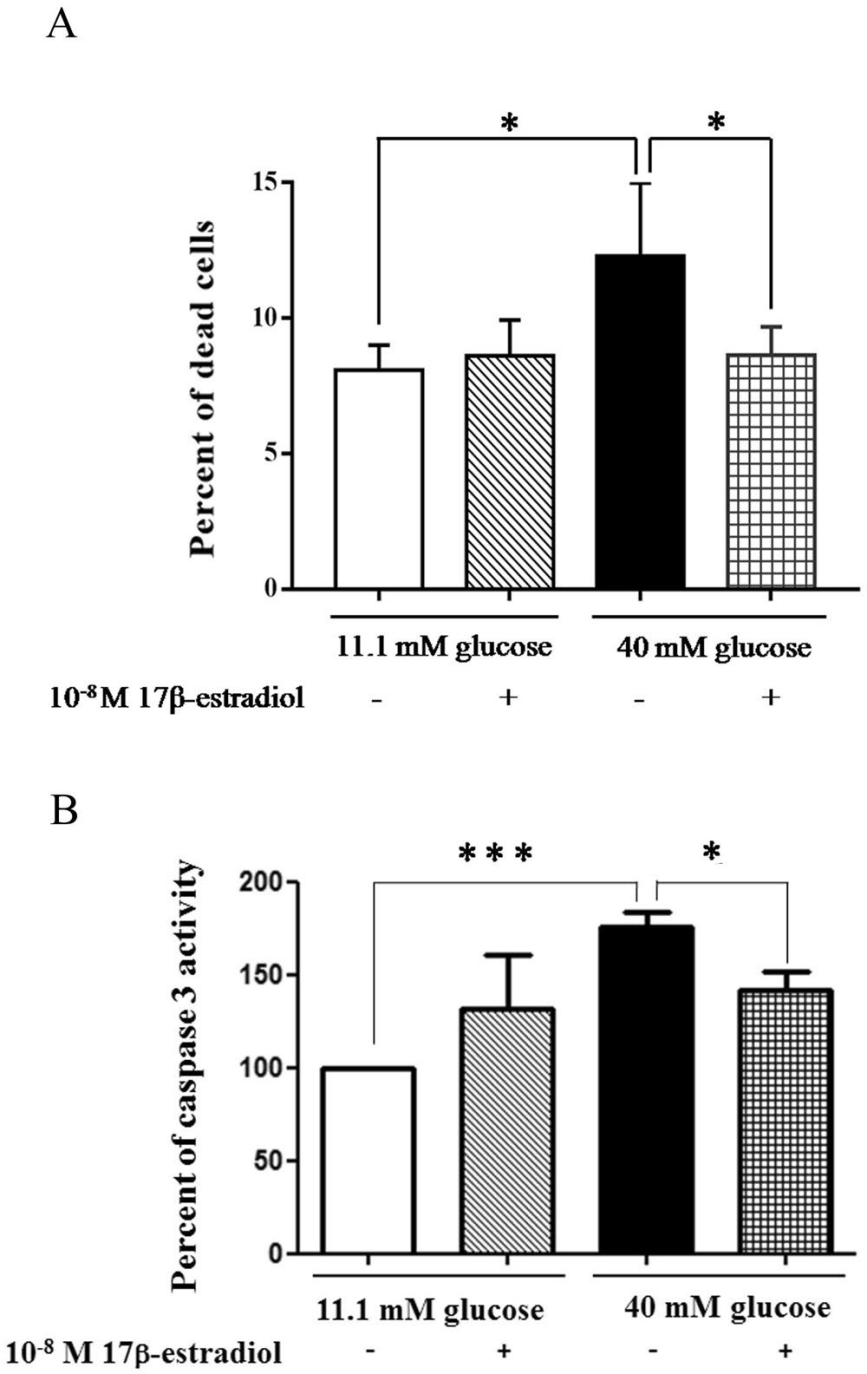
Cell death was measured using FACS to analyze PI staining and caspase 3 activity. (**A**) The percentage cell death in INS-1 cells cultured in experimental conditions. (**B**) Caspase 3 activity in INS-1 cells cultured in experimental conditions. The mean ± S.D. of 4 independent experiments are shown. *P < 0.05 and ***P < 0.001.

To investigate the effect of estrogen on INS-1 cell death, cells were cultured in basal or high glucose media, with or without 10^−8^ M 17β-estradiol. Dead cells were detected by propidium iodine (PI) staining, and were analyzed by flow cytometer. The total percentage of dead cells was significantly increased in INS-1 cells cultured in the high glucose medium compared to those cultured in the basal glucose medium. However, the presence of 10^−8^ M 17β-estradiol in the INS-1 cells cultured in the high glucose medium significantly reduced the percentage of cell deaths (Fig. 1A). In contrast, the addition of 10^−8^ M 17β-estradiol to the INS-1 cells cultured in the basal glucose medium showed no difference in the proportion of cell deaths compared to the proportion for the INS-1 cells cultured in the basal glucose medium alone.

To confirm cell death, the caspase 3 activities were measured in INS-1 cells cultured in basal or high glucose media, with or without 10^−8^ M 17β-estradiol. As expected, the caspase 3 activity was significantly increased when the INS-1 cells were cultured in a high glucose medium compared to INS-1 cells cultured in a basal glucose medium. The addition of 10^−8^ M 17β-estradiol to the INS-1 cells cultured in a high glucose medium significantly reduced the caspase3 activity compared to when the INS-1 cells were cultured only in a high glucose medium (Fig. 1B). These results indicated that a high glucose medium increased β-cell death, which was ameliorated by the estrogen treatment.

### Estrogen decreased AGTR1 mRNA and protein expression in INS-1 cells cultured in high glucose medium

**Figure 2:**
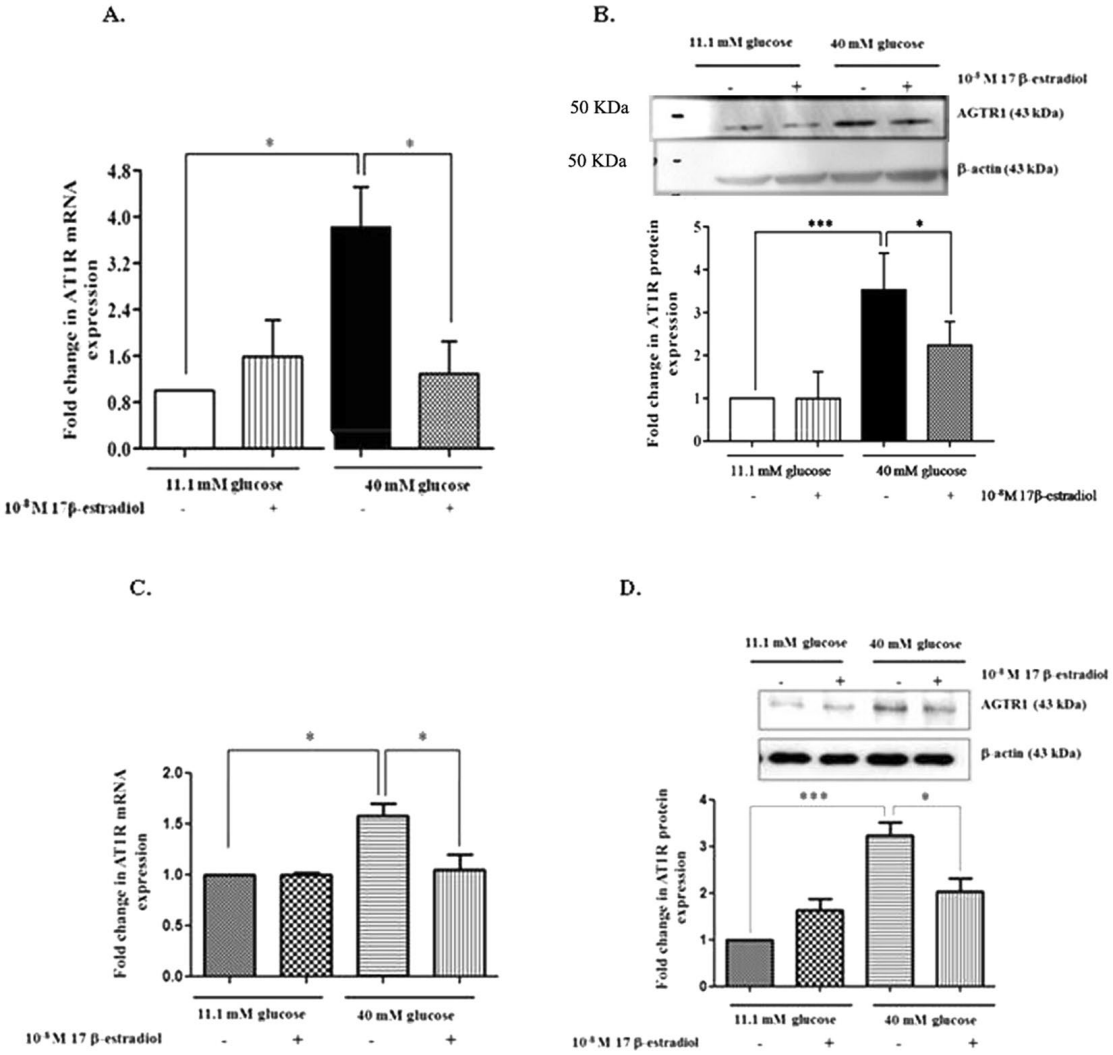
*AGTR1* mRNA and protein expression from INS-1 cells and mouse isolated pancreatic islets that were cultured in normal and high glucose. (**A**) Fold change of *AGTR1* mRNA from INS-1 cells as normalized to *β-actin* mRNA at 48 hrs after treatment with high glucose media. (**B**) Above picture is a representative Western blot of *AGTR1* and β-actin proteins from INS-1 cells. The bar graph below demonstrates fold change in *AGTR1* protein normalized to β-actin protein. (**C**) Fold change of *AGTR1* mRNA from isolated islets as normalized to *β-actin* mRNA at 10 days after treatment with high glucose media. (**D**) Above picture is a representative Western blot of *AGTR1* and β-actin proteins from isolated islets. The bar graph below demonstrates fold change in *AGTR1* protein normalized to β-actin protein. The data are presented as the mean ± S.D. of 3–4 independent experiments. *P < 0.05 and **P < 0.01. All full-length blots are presented in Supplementary Figure 1.

We further examined the effect of estrogen on *AGTR1* mRNA expression by real-time PCR. Our results showed that *AGTR1* mRNA expression was significantly increased in INS-1 cells cultured in a high glucose medium compared with those cultured in a basal glucose medium (Fig. 2A). The addition of 10^−8^ M 17β-estradiol in INS-1 cells cultured in a high glucose medium significantly reduced *AGTR1* mRNA expression, compared to those cultured in a high glucose medium alone. In contrast, 10^−8^ M 17β-estradiol supplementing INS-1 cells cultured in a basal glucose medium did not have any effect on *AGTR1* mRNA levels, compared to those cultured in just a basal glucose medium. This finding suggests that estrogen down-regulates the *AGTR1* mRNA expression in high glucose conditions.

To determine whether estrogen also decreased *AGTR1* protein levels, the *AGTR1* protein levels from experimental conditions were determined by Western blot analysis. INS-1 cells cultured in a high glucose medium significantly increased *AGTR1* protein expression (by approximately 3.53 fold), compared with those cultured in a basal glucose medium. However, a supplement of 10^−8^ M 17β-estradiol added to the INS-1 cells cultured in a high glucose medium significantly reduced the *AGTR1* protein expression (Fig. 2B). On the other hand, there was no difference in the *AGTR1* protein expression in INS-1 cells cultured in a basal glucose medium, with or without the addition of 10^−8^ M 17β-estradiol.

### Estrogen reduced AGTR1 mRNA and protein expression in pancreatic β-cells cultured in high glucose medium

To verify whether estrogen attenuates *AGTR1* expression in *ex vivo*, *AGTR1* mRNA and protein expressions in pancreatic islets cultured in basal and high glucose media, and with or without 10^−8^ M 17β-estradiol, were assessed by real-time PCR and Western blot analysis. Similar to previous results in INS-1 cells, *AGTR1* mRNA and protein expressions significantly increased in pancreatic islets cultured with a high glucose medium compared to the control. The presence of 10^−8^ M 17β-estradiol in pancreatic islets cultured with a high glucose medium markedly reduced the *AGTR1* mRNA levels and protein expressions, compared to those cultured only in a high glucose medium. By comparison, there was no significant change in *AGTR1* mRNA levels and protein expressions in pancreatic islets cultured in basal glucose media, with or without 10^−8^ M 17β-estradiol (Fig. 2C and D).

### Estrogen, but not losartan, decreased AGTR1 protein expression in INS-1 cells cultured in high glucose medium with AngII

**Figure 3:**
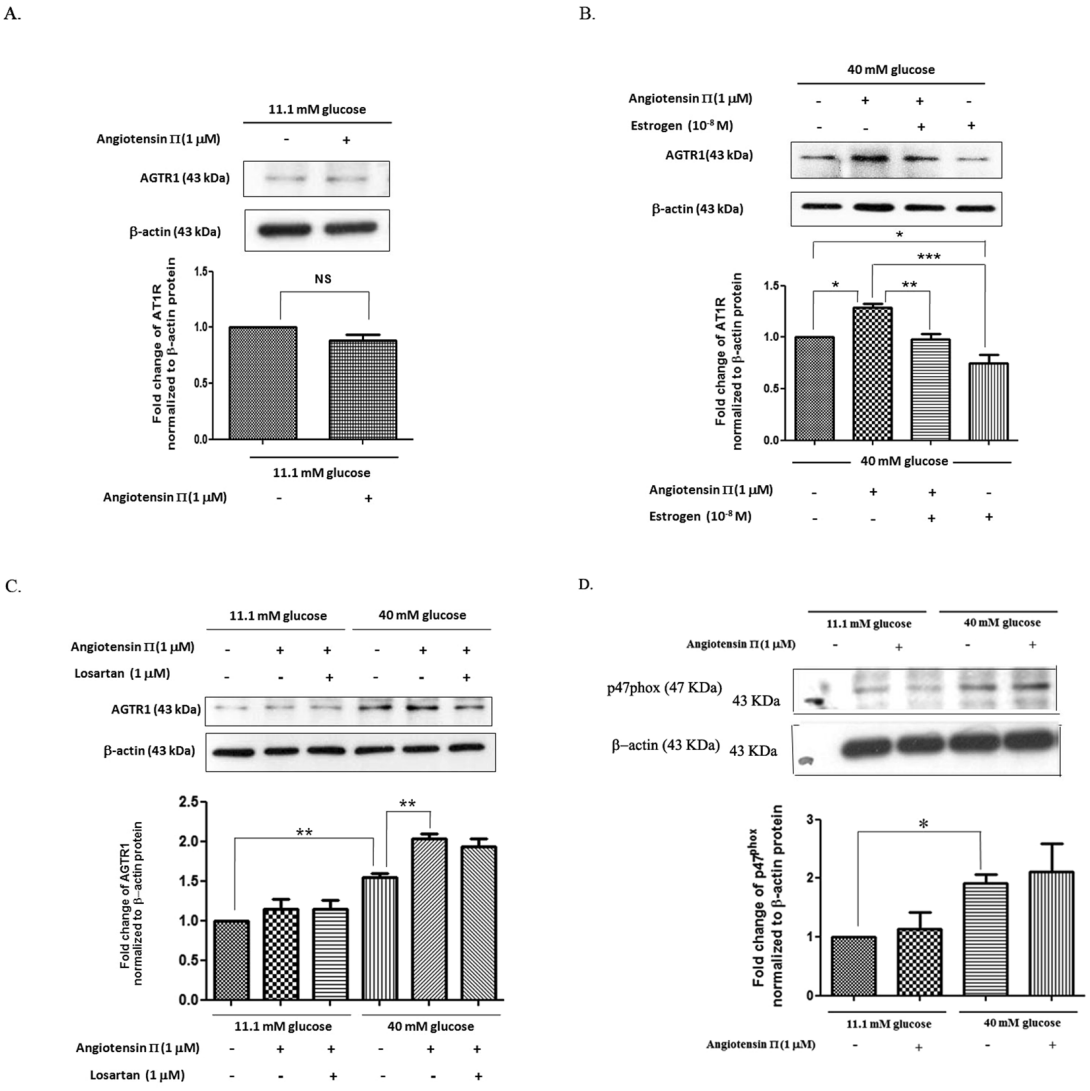
Effects of basal & high glucose media, Ang II and estrogen on *AGTR1* protein expressions in INS-1 cells. (**A**) INS-1 cells were cultured with basal glucose medium, with or without 1 μM Ang II, for 72 h. Above picture is a representative Western blot of *AGTR1* and β-actin proteins. The bar graph below demonstrates fold change in *AGTR1* protein normalized to β-actin protein. (**B**) INS-1 cells were cultured in high glucose medium in the present or absent 1 μM Ang II, with or without 10^−8^ M 17β-estradiol, for 72 h. Above picture is a representative Western blot of *AGTR1* and β-actin proteins. The bar graph below shows fold change in *AGTR1* protein to control group, and normalized to β-actin protein. (**C**) INS-1 cells were cultured with basal glucose or high glucose medium, with or without 10^−8^ M 17β-estradiol, in the presence or absence of 10 μM losartan. Above picture is a representative Western blot of *AGTR1* and β-actin proteins. The bar graph below shows fold change in *AGTR1* protein to control group, and normalized to β-actin protein. (**D**) INS-1 cells were cultured with basal glucose or high glucose medium, with or without 1 μM Ang II. Above picture is a representative Western blot of p^47phox^ and β-actin proteins. The bar graph below shows fold change in p^47phox^ protein to control group, and normalized to β-actin protein. All data are expressed as mean ± S.D. of 3 independent experiments. ^*^ *P* < 0.05, ^**^ *P* < 0.01, ^***^ *P* < 0.001. NS is not significant. All full-length blots are presented in Supplementary Figure 3.

To examine whether AngII has an effect on *AGTR1* expression in basal and high glucose media, AngII was added to INS-1 cells cultured in basal and high glucose media. The results indicated that AngII had no effect on the AT1R expression in the basal glucose medium, but AngII increased the AT1R expression in the high glucose medium (Fig. 3A). We further investigated whether estrogen reduced the AGTR 1 expression in the presence of AngII in a high glucose medium. Our results showed that AngII cultured with a high glucose medium significantly increased the AT1R expression. Estrogen cultured with a high glucose medium alone, or cultured with AngII in a high glucose medium, reduced the AGTR 1 expression (Fig. 3B). Lastly, we further examined whether losartan could reduce the AGTR 1 expression in the presence of AngII in a basal or a high glucose medium. The results showed that losartan did not decrease the AGTR 1 expression in the presence of AngII in either a basal or a high glucose medium (Fig. 3C). The addition of AngII in basal glucose had a trend to increase *p47* ^*phox*^ expression when compared with basal glucose condition. AngII in high glucose has no additive effect on *p47* ^*phox*^ expression when compared with high glucose alone. AngII showed a similar pattern of expression in both AGTR1 and *p47* ^*phox*^ (Fig. 3D).

### Estrogen decreased AGTR1 downstream signaling in INS-1 cells cultured in high glucose medium

**Figure 4:**
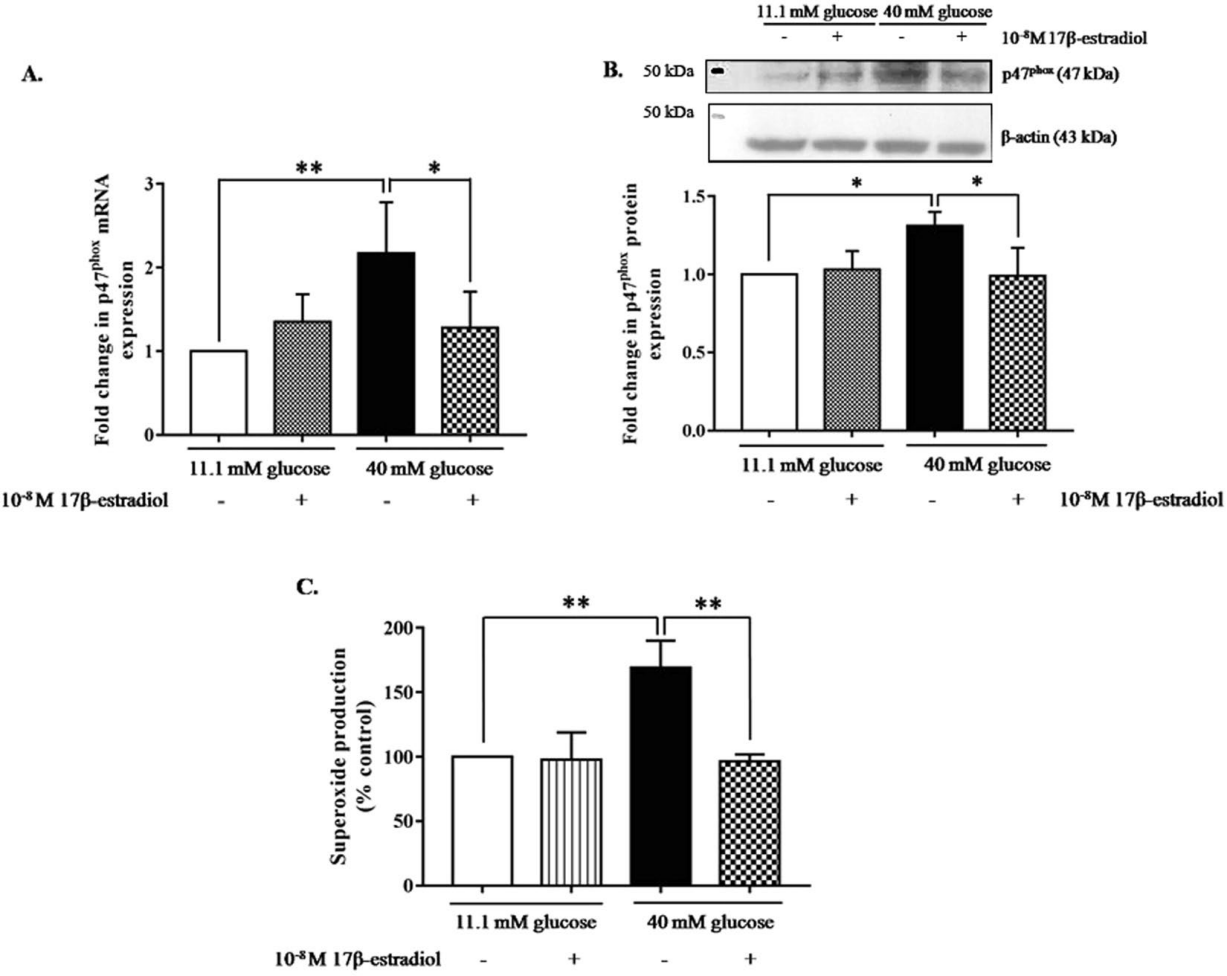
*p47* ^*phox*^ mRNA and protein expression analysis and superoxide production. INS-1 cells were cultured in normal and high glucose media, with or without 10^−8^ M 17β-estradiol. (**A**) Fold change *p47* ^*phox*^ mRNA normalized to *β-actin* mRNA. (**B**) Above picture is a representative Western blot of *p47* ^*phox*^ and β-actin proteins. The bar graph below shows fold change in *p47* ^*phox*^ protein to control group, and normalized to β-actin protein. (**C**) Superoxide production was measured using NBT assay. Amount of superoxide was determined by optical density (OD). The bar graph shows production of superoxide in INS-1 cells. The data are presented as the mean ± S.D. of 4 independent experiments. *P < 0.05 and **P < 0.01. All full-length blots are presented in Supplementary Figure 4.

We further examined the role of estrogen in *AGTR1* downstream signaling by measuring a NADPH oxidase enzyme subunit, *p47* ^*phox*^, mRNA and protein expression. A high glucose medium significantly increased the *p47* ^*phox*^ mRNA expression compared to those cultured in a basal glucose medium. As expected, 10^−8^ M 17β-estradiol co-cultured with a high glucose medium brought the *p47* ^*phox*^ mRNA expression back to a similar level as in the control condition. There were no changes in the *p47* ^*phox*^ mRNA expressions in the INS-1 cells cultured in a basal glucose medium either in the presence or the absence of 10^−8^ M 17β-estradiol (Fig. 4A). These results indicate that 10^−8^ M 17β-estradiol down-regulates *p47* ^*phox*^ mRNA expression in high glucose conditions.

To verify whether estrogen decreases *p47* ^*phox*^ protein levels, the levels of *p47* ^*phox*^ protein from INS-1 cells cultured in experimental conditions were determined by Western blot analysis. Similar to the mRNA expression, the *p47* ^*phox*^ protein expression increased significantly in INS-1 cells that were cultured in high glucose conditions and was reduced markedly by estrogen treatment. There were no differences in the *p47* ^*phox*^ protein expressions in INS-1 cells cultured in basal glucose conditions, with or without 10^−8^ M 17β-estradiol (Fig. 4B). These results confirm that estrogen reduced *AGTR1* downstream mRNA levels and protein expressions in high glucose conditions.

### Estrogen decreased oxidative stress in INS-1 cells cultured in high glucose medium

NADPH oxidase complexes produce superoxide as a downstream product of *AGTR1* pathway activation. We examined whether estrogen reduced NADPH oxidase activity in INS-1 cells cultured in a high glucose medium. The superoxide anion in INS-1 cells cultured in each experimental condition was measured by an NBT assay. The superoxide production was increased significantly in the INS-1 cells cultured in a high glucose medium, compared with those cultured in a basal glucose medium. Estrogen added to INS-1 cells cultured in a high glucose medium lowered the superoxide production relative to the control level. In comparison, the addition of estrogen to INS-1 cells cultured in a basal glucose medium had no effect on superoxide production, relative to the control (Fig. 4C).

### Estrogen and losartan increased cell viability in INS-1 cells cultured in high glucose medium

**Figure 5:**
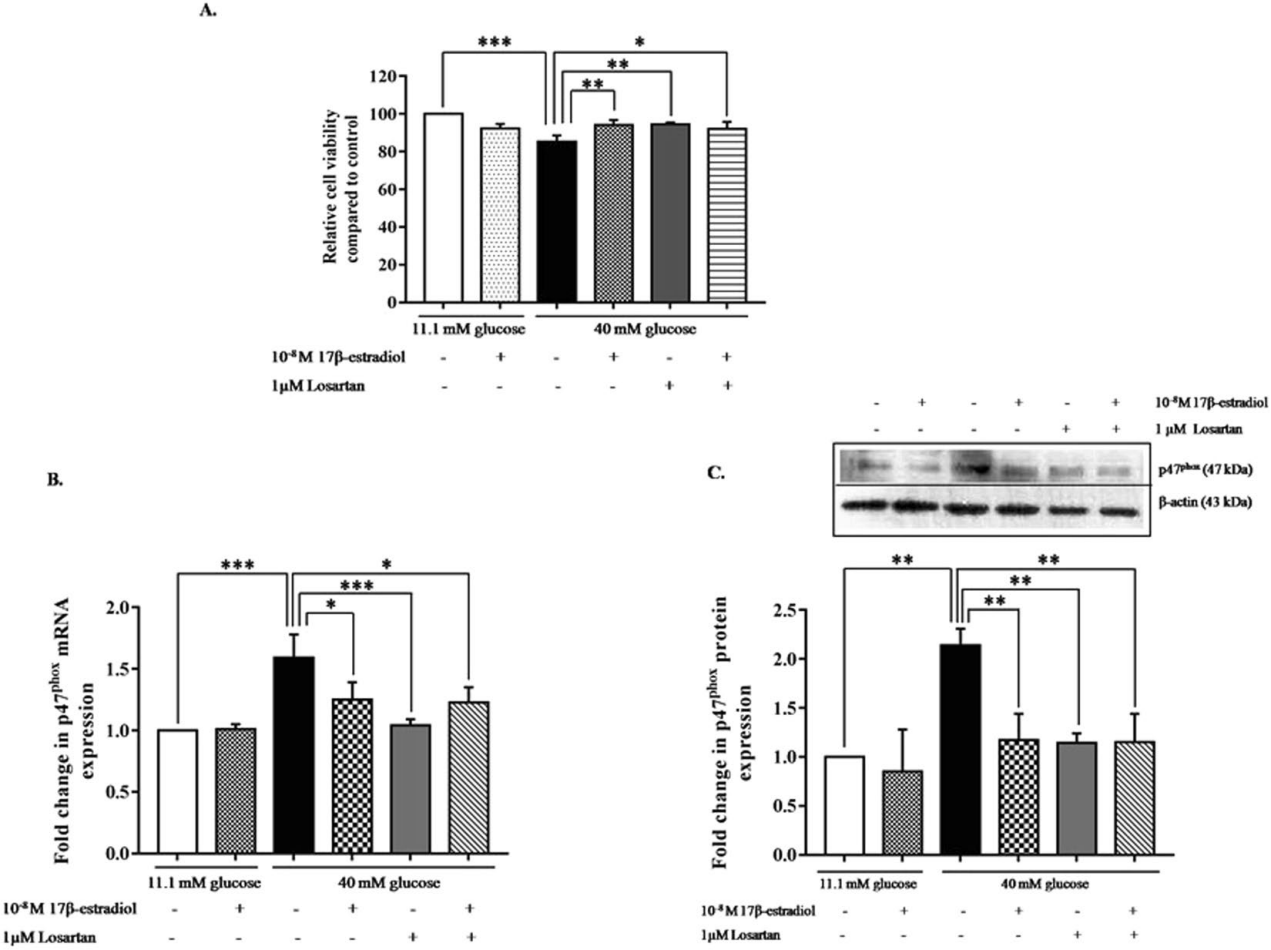
Cell viability and *p47* ^*phox*^ mRNA and protein expression from INS-1 cells cultured in basal and high glucose media in the presence or absence of estrogen with or without losartan. (**A**) Cell viability was measured by MTT assay. Each condition was performed in triplicate for each experiment. (**B**) *p47* ^*phox*^ mRNA and protein expression from INS-1 cells cultured in basal and high glucose media, with or without 10^−8^ M 17β-estradiol, or 1μM losartan alone, or in combination. (**C**) A. Fold change of *p47* ^*phox*^ mRNA as normalized to *β-actin* mRNA. *P < 0.05 and **P < 0.01. (**B**) Representative Western blot analysis of *p47* ^*phox*^ protein as normalized to β-actin protein. The data are presented as the mean ± S.D. of 3–4 independent experiments. *P < 0.05, **P < 0.01and ***P < 0.001. All full-length blots are presented in Supplementary Figure 5.

To investigate whether estrogen and the angiotensin II receptor blocker, losartan, have a similar effect on cell viability, the viability was assessed in INS-1 cells cultured in basal and high glucose media, with or without 10^−8^ M 17β-estradiol, 1 µM losartan, or a combination of both. Approximately 85% of the cells in the high glucose medium were viable compared with those that were cultured in a normal glucose medium. When, cells that were cultured in a high glucose medium were treated with 10^−8^ M 17β-estradiol, the percentage of viable cells increased significantly to 94%, which was similar to those that were treated with 1 µM losartan, or with a combination of both 10^−8^ M 17β-estradiol and 1 µM losartan. In contrast, the percentage of viable cells did not differ for INS-1 cells cultured in a basal glucose medium with 10^−8^ M 17β-estradiol, 1 µM losartan, or a combination of both (Fig. 5A). These results suggested that both estrogen and losartan increased cell survival to similar levels under high glucose conditions.

### Estrogen and losartan decreased *p47*^*phox*^ mRNA and protein expression in INS-1 cells cultured in high glucose medium

To investigate whether estrogen and losartan act through the *AGTR1* signaling pathway, *p47* ^*phox*^ mRNA and protein expression were measured in INS-1 cells that were cultured in normal and high glucose media, with or without estrogen or losartan alone or in combination. The *p47* ^*phox*^ mRNA expression was increased significantly under high glucose conditions, whereas treatment with estrogen, losartan, or both in combination reduced *p47* ^*phox*^ mRNA expression significantly. To determine whether *p47* ^*phox*^ protein levels were also reduced, Western blots were performed. It was determined that *p47* ^*phox*^ protein levels decreased under high glucose conditions, similar to the mRNA levels. Treatment with estrogen, losartan, or a combination of both significantly reduced *p47* ^*phox*^ protein expression, and to a similar extent. These results suggest that estrogen and losartan have no additive or synergistic effects to reduce *p47* ^*phox*^ mRNA and protein expression (Fig. 5B and C). As well, the results indicate that estrogen and losartan ameliorate pancreatic β-cell death by reducing the *AGTR1* pathway.

## Discussion

Chronic hyperglycemia has been observed as a pathological cause of pancreatic β-cell death in type 2 diabetic patients, which is known as glucotoxicity^3,21^. The mechanism of glucotoxicity has been investigated over several decades. A number of studies have proposed that the central mechanism of glucotoxicity is the overproduction of oxidative stress in pancreatic β-cells^3,4,6,21^. This oxidative stress occurs due to the increased production of reactive oxygen species (ROS) or reactive nitrogen species (RNS)^22^. Local angiotensin II (Ang II) produces ROS by AGTR activation^23^. The components of RAS are found in both human and murine pancreatic islets^24,25^. The protective effect of estrogen against diabetes has been reported in several studies^26–29^. However, the effect of estrogen on local pancreatic RAS has not been studied.

This study showed that a high glucose medium increased pancreatic cell death, *AGTR1* mRNA levels and protein expression. Our results were comparable to the findings from another study in which a high glucose medium induced *AGTR1* mRNA and protein expression^12^. Local RAS has been proposed as a pathological process to induce pancreatic β-cell death via oxidative stress^30^. Our previous study showed that knock down *AGTR1* reduced caspase 3 expression in a high glucose medium^31^, which suggested that high glucose might induce pancreatic β-cell apoptosis via the local pancreatic RAS. The local RAS has been demonstrated as a pathological process in diabetic complication^32–34^. For example, high glucose activated the RAS by increasing Ang II and AT1R expression, which causes damage to, and the loss of, podocytes in diabetic nephropathy^35^. RAS also plays an important role in the pathogenesis of atherosclerosis^36^.

It has been proposed that high glucose increases AngII production in rodent pancreatic β-cells^37^ and human islets^38^. Thus, it is possible that AngII might act as a local hormone and induce pancreatic β-cell death. Our previous study showed that AngII cannot increase the percentage of pancreatic β-cell apoptosis in basal glucose conditions, and AngII slightly increased the proportion of pancreatic β-cell apoptosis in high glucose conditions^31^. This suggested that AngII required upregulated *AGTR1* expression to induce pancreatic β-cell death. This study examined this notion by adding AngII in experimental conditions. Our results showed that AngII did not increase *AGTR1* expression in a basal glucose medium, but AngII significantly stimulated *AGTR1* expression in high glucose conditions. On the other hand, estrogen significantly decreased *AGTR1* expression in a high glucose medium, with or without AngII. This is evidence that *AGTR1* expression plays an important role in inducing pancreatic β-cell apoptosis. This notion corresponded with the findings of several studies that a blocker of the pancreatic local RAS can prevent pancreatic β-cell death both *in vitro* and *in vivo* ^10,11,39,40^.

Estrogen in a high glucose medium significantly decreased pancreatic β-cell death and *AGTR1* mRNA and protein expression in INS-1 cells. The effect of estrogen in a high glucose medium was also examined using mouse pancreatic islets. A similar result was shown: that estrogen reduced *AGTR1* mRNA levels and protein expression in the mouse pancreatic islets. It is known that activated *AGTR1* induces NADPH oxidase enzyme complexes, which consist of two membrane-bound subunits (*gp91* ^*phox*^ and *p22* ^*phox*^), three cytosolic subunits (*p40* ^*phox*^, *p47* ^*phox*^ and *p67* ^*phox*^), and a low molecular weight G-protein (Rac2 or Rac1)^41^. This study further examined the effect of high glucose and estrogen on *p47* ^*phox*^, an *AGTR1* downstream protein. Our results showed that a high glucose medium increased *p47* ^*phox*^ mRNA and protein expression, whereas estrogen co-cultured with high glucose brought *p47* ^*phox*^ mRNA and protein expression back to levels similar to those in the control condition. Our results were comparable with those from other studies that reported an upregulation of NADPH oxidase subunits *p47* ^*phox*^, *gp91* ^*phox*^ and *p22* ^*phox*^ in diabetic animal rodents^42^. Altogether, estrogen seems to reduce both *AGTR1* and its downstream signaling protein. Activated NADPH oxidase produces ROS in cells. To verify NADPH oxidase enzyme activity, the superoxide production in each experimental condition was measured. Again, a high glucose medium increased superoxide production, but the presence of estrogen with a high glucose medium reversed this effect. Estrogen-inhibited NADPH oxidase expression and activity has also been reported in endothelial and monocyte cells^43,44^. Although, this study did not measured AngII production in the medium, previous study showed that local RAS enzymes, renin, angiotensinogen and ACE, were up-regulated by high glucose^9^. Whether estrogen decreased local AngII production is still unknown. There is an *in vivo* study demonstrated that estrogen replacement in ovariectomized rat reduced plasma ACE and AngII levels^45^. Whether estrogen decreased local pancreatic AngII production is required further investigation.

The study compared the protective effects of estrogen and the *AGTR1* inhibitor, losartan, on high-glucose-induced pancreatic β-cell death. Our results demonstrated that estrogen and losartan reduced pancreatic β-cell death and *p47* ^*phox*^ expression. Estrogen and losartan did not have an additive effect to protect against high-glucose-induced pancreatic β-cell death. It is possible that estrogen and losartan might exploit similar pathways. The effect of estrogen on the inhibition of *AGTR1* expression has been shown in several tissues, including vascular smooth muscle^16^, the heart^18^ and the adrenal glands^17^. However, it is worth noting that estrogen has been shown to reduce pancreatic β-cell apoptosis through several pathways, including an antioxidant effect^18,29^ and the reduction of ER stress^26^. Estrogen inhibited-*AGTR1* expression might be another pathway by which estrogen prevents pancreatic β-cell apoptosis due to high glucose levels. For a future direction, these results should be verified in animal model of type 2 diabetes. The molecular mechanisms of estrogen on *AT1R* down-regulation should be explored, which should be tested for both genomic and non-genomic pathways. The better understanding of these mechanisms may lead to the identification of novel substances for treatment of diabetes.

In conclusion, we demonstrated that estrogen reduces high-glucose-induced pancreatic β-cell death through the reduction of the *AGTR1* pathway both in cell line and rodent islets. The molecular mechanism of estrogen-inhibiting *AGTR1* activation was not revealed in this study. Identification of the molecular mechanism by which estrogen decreases the *AGTR1* pathway merits further investigation.

## Electronic supplementary material

Estrogen attenuates AGTR1 expression to reduce pancreatic β-cell death from high glucose.
