# Supplementary material for: Estrogen attenuates AGTR1 expression to reduce pancreatic β-cell death from high glucose
Source: Sci Rep. 2017 Nov 30;7:16639. doi: 10.1038/s41598-017-15237-4 (PMC5709427; doi:10.1038/s41598-017-15237-4)
Supplement: Supplementary file 1 — Estrogen attenuates AGTR1 expression to reduce pancreatic β-cell death from high glucose. [file 41598_2017_15237_MOESM1_ESM.pdf]

Estrogen attenuates *AGTR1* expression to reduce pancreatic β-cell death from high glucose.

Suwattanee Kooptiwut*[1], Keerati Wanchai[1], Namoiy Semprasert[1], Chatchawan Srisawat[2], Pa-thai Yenchitsomanus[4]

[1] Department of Physiology, [2] Department of Biochemistry, [3] Department of Anatomy, [4] Department of Research and Development (Division of Molecular Medicine), Faculty of Medicine, Siriraj Hospital, Mahidol University, Bangkok 10700, Thailand

Address of corresponding author:

Suwattanee Kooptiwut, M.D., Ph.D.,

Department of Physiology, 2 Prannok,

Faculty of Medicine, Siriraj Hospital, Mahidol University,

Bangkok 10700, Thailand,

Tel: 66-2-419720; Fax: 66-2-4115009

Email addresses: siskw@mahidol.ac.th, S_kooptiwut@hotmail.com

Original blot figure (Figure 2B)

AT1R (43 kDa):

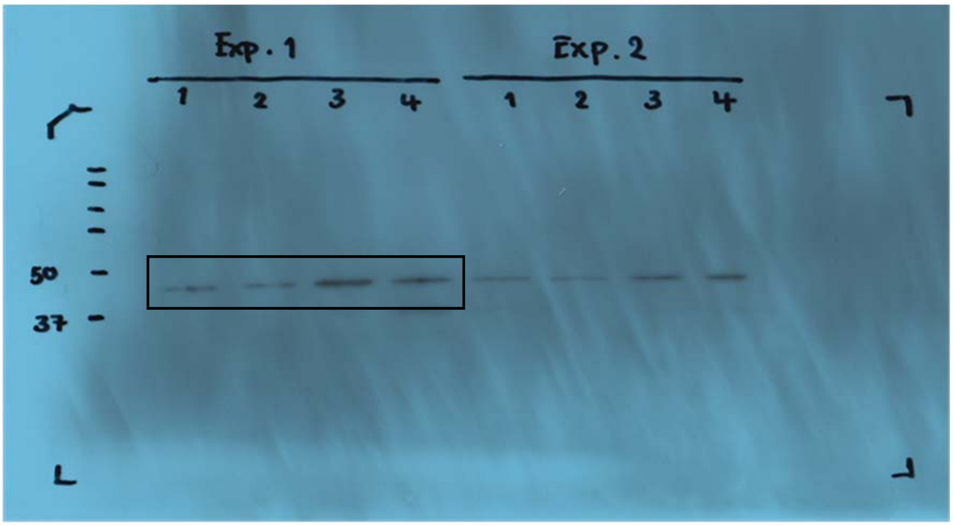

β-actin (43 kDa):

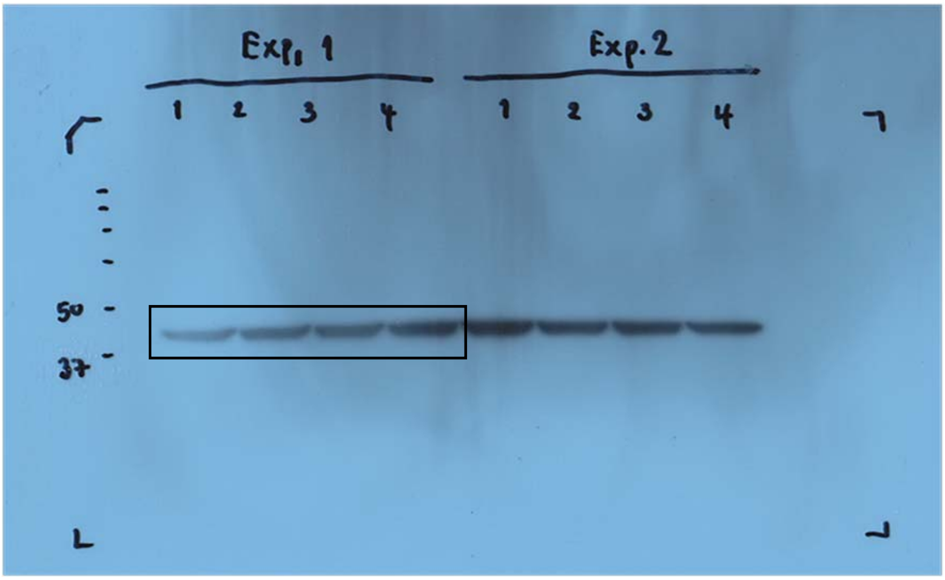

Supplement figure 1

Original blot figure (Figure 2D)

AT1R (43 kDa):

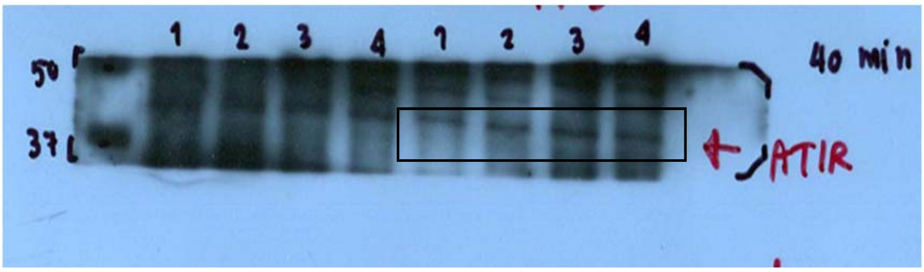

β-actin (43 kDa):

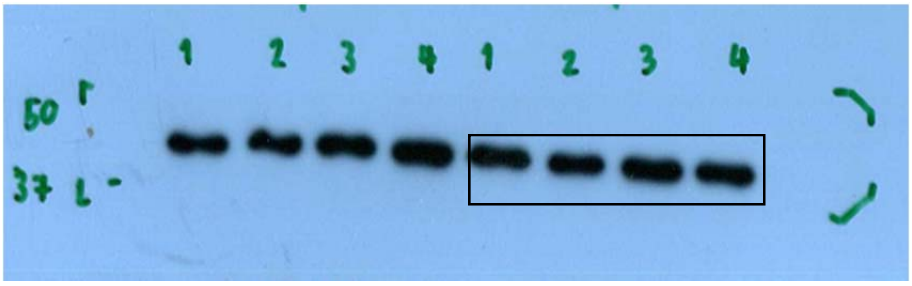

Supplement figure 2

Original blot figure (Figure 3A, B, C&D)

AT1R (43 kDa):

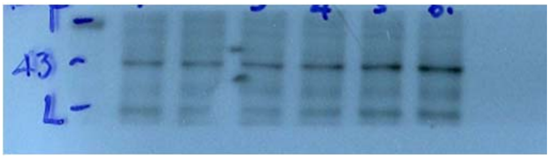

β-actin (43 kDa):

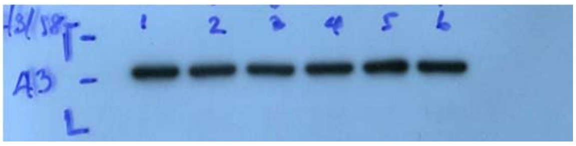

(Figure 3B)

AT1R (43 kDa):

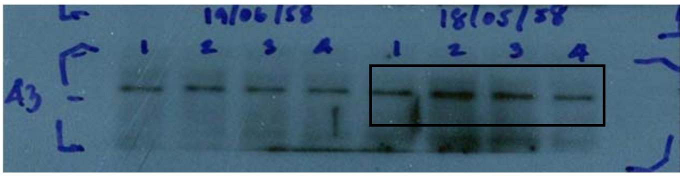

β-actin (43 kDa):

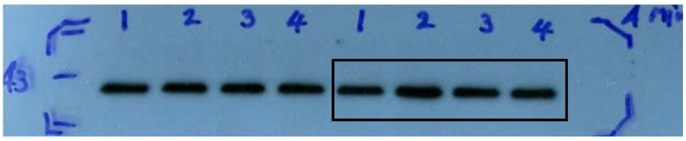

(Figure 3C)

AT1R (43 kDa):

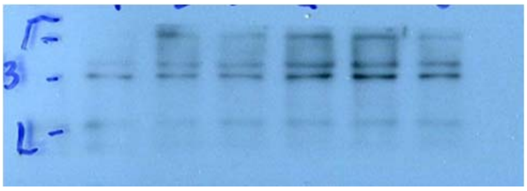

β-actin (43 kDa):

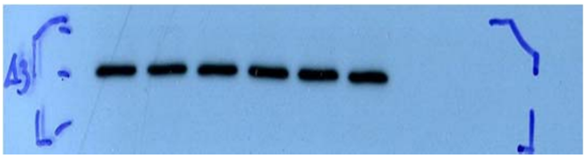

Supplement figure 3

(Figure 3D)

p[47phox] (47 kDa):

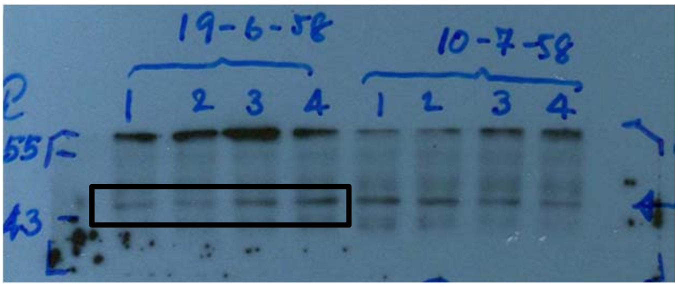

β-actin (43 kDa):

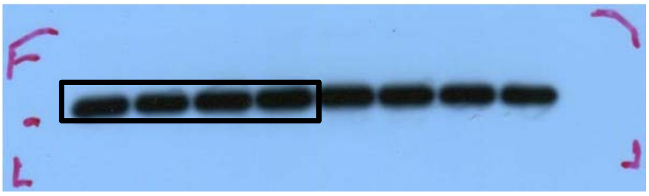

Supplement figure 3

Original blot figure

Figure 4B

p^{47phox} (47 kDa):

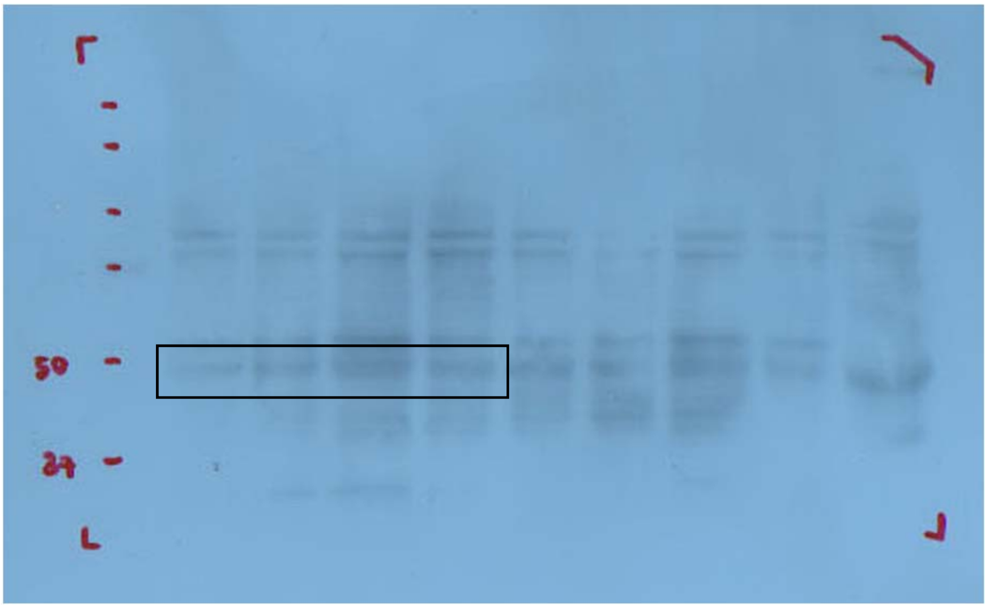

β-actin (43 kDa):

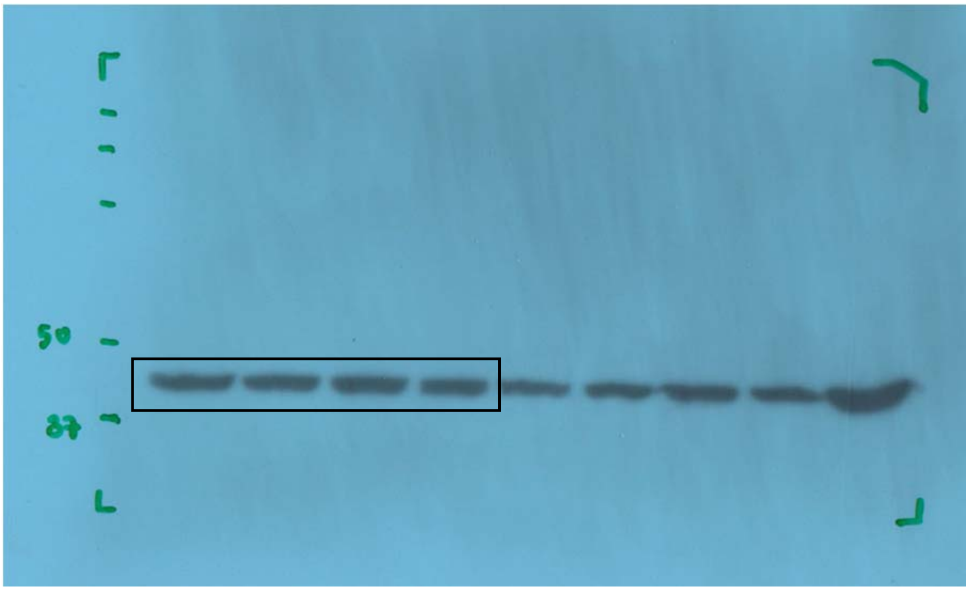

Supplement figure 4

Original blot figure

Figure 5C

p^{47phox} (47 kDa):

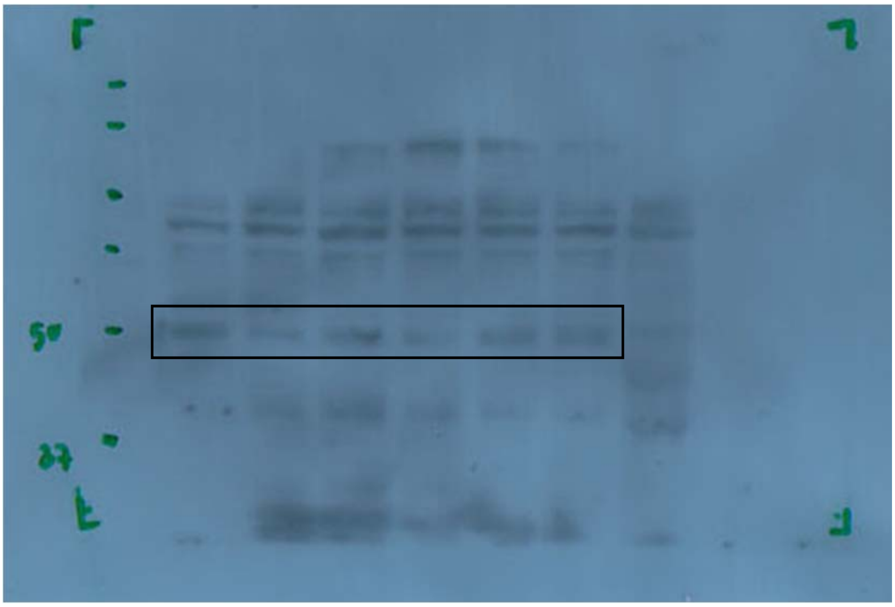

β-actin (43 kDa):

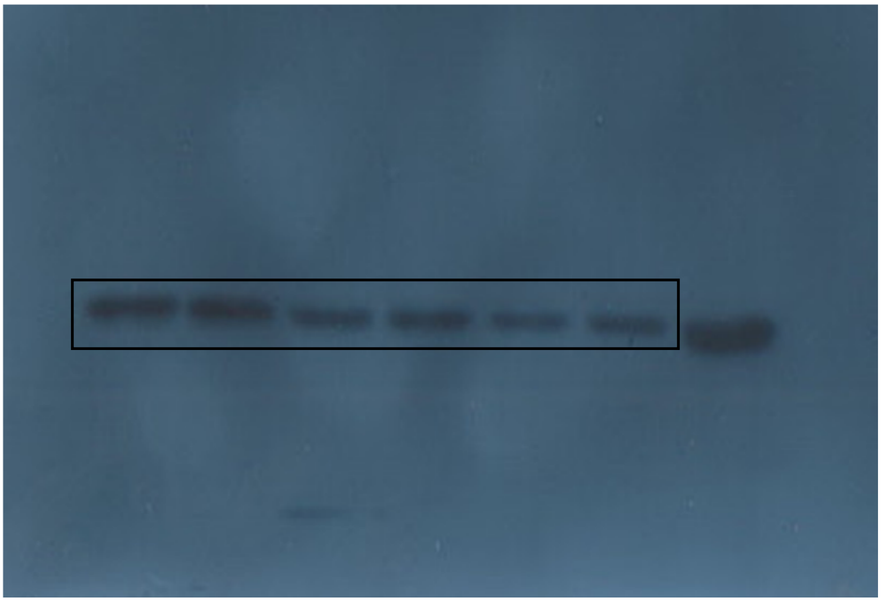

Supplement figure 5
